# Anesthetic management of an infant with Pierre Robin sequence undergoing tracheostomy using an i-gel™

**DOI:** 10.1186/s40981-019-0231-4

**Published:** 2019-02-19

**Authors:** Keisuke Omiya, Takashi Matsukawa

**Affiliations:** Department of Anesthesiology, Faculty of Medicine, University of Yamanashi, 1110 Shimokato Chuo-shi, Yamanashi, 409-3898 Japan

**Keywords:** Pierre Robin sequence, I-gel™, Tracheostomy, Infant

To the Editor:

Pierre Robin sequence (PRS) is defined as the clinical triad of micrognathia, glossoptosis, and airway obstruction. Proper airway management is necessary during infancy because children with PRS frequently experience respiratory failure in early life [1]. There are some reports that a laryngeal mask airway (LMA) and the Air-Q® (Mercury Medical, Clearwater, FL, USA) are effective for airway management in patients with PRS [2, 3]. There are also some reports that the i-gel™ (Intersurgical, Wokingham, UK) is more effective than an LMA because inserting an i-gel™ takes less time than inserting an LMA [4, 5]; furthermore, the i-gel™ has greater leak pressure than the LMA [5]. However, there are no case reports regarding tracheostomy of infants with PRS, especially young infants approximately 1 month old, using an i-gel™. Therefore, we evaluated the use of the i-gel™ as a supraglottic airway device (SGA) during this procedure.

The patient was a 46-day-old boy with a height of 49 cm and a weight of 2.6 kg. He was born at 37 weeks and weighed 1970 g. He was diagnosed with PRS because he had micrognathia and a cleft palate at birth. The patient’s pharyngeal view was categorized as Mallampati class IV. It was possible to fit a mask to his face. A tracheostomy was scheduled because of worsening airway obstruction.

Preanesthetic medication was not administered. An intravenous catheter was placed before the patient entered the operating room. Electrocardiography, percutaneous oxygen saturation, and noninvasive arterial blood pressure were monitored after the patient entered the operating room. We induced general anesthesia after preoxygenating the patient with 5 L/min oxygen for 3 min. First, we administered 0.06 mg intravenous injection (IV) atropine, 6 μg IV fentanyl, and 6 mg IV propofol. We were able to provide ventilation via a mask; subsequently, we administered 3 mg of IV rocuronium. We then attempted to intubate the patient with an uncuffed endotracheal tube (3.0 mm internal diameter) using an AirWay Scope™ (AWS™, Pentax, Humburg, DE). However, we could not visualize the vocal cords (Cormach-Lehane grade 4). We knew that attempts at tracheal intubation frequently lead to laryngeal edema and the inability to provide ventilation. Therefore, we inserted an i-gel™ (size1). We were able to provide ventilation via the i-gel™, and thought that it would be possible to perform a tracheostomy if we could provide ventilation via the i-gel™ using rocuronium to open the glottis. Anesthesia was maintained with a gas mixture of oxygen, air (fraction of inspired oxygen (FIO_2_), 0.3–0.7) and sevoflurane (1–1.5%), and remifentanil (0.1–0.5 μg/kg/min) and rocuronium (single injection of 1.5 mg) were administered. We were able to perform a tracheostomy and surgery without complications.

We successfully used an i-gel™ for anesthetic management of an infant with PRS who was undergoing tracheostomy.

## Abbreviations

AWS™: AirWay Scope™
IV: Intravenous injection
LMA: Laryngeal mask airway
PRS: Pierre Robin sequence
SGA: Supraglottic airway device

## Authors’ information

The corresponding author’s secondary institution is Shizuoka Children’s Hospital, Japan.
